# Loss of scinderin decreased expression of epidermal growth factor receptor and promoted apoptosis of castration‐resistant prostate cancer cells

**DOI:** 10.1002/2211-5463.12412

**Published:** 2018-04-10

**Authors:** Xiaofeng Lai, Weipeng Su, Hu Zhao, Shunliang Yang, Tengyue Zeng, Weizhen Wu, Dong Wang

**Affiliations:** ^1^ State Key Laboratory of Cancer Biology Department of Biochemistry and Molecular Biology The Fourth Military Medical University Xi'an China; ^2^ Department of Urology Fuzhou General Hospital Fujian Medical University Fuzhou China; ^3^ Department of Urology Fuzhou General Hospital (Dongfang Hospital) Xiamen University Fuzhou China

**Keywords:** actin cytoskeleton, apoptosis, epidermal growth factor receptor, prostate cancer, scinderin

## Abstract

Most patients with prostate cancer will eventually develop the castration‐resistant form characterised by metastasis. Cytoskeleton constituents, including F‐actin, play important roles in maintaining epithelial integrity and their disruption is a major cause of cancer progression. We previously showed that scinderin (SCIN), an important regulator of F‐actin organisation, is highly expressed in poorly differentiated cancer tissues. This study aimed to explore the mechanism of its regulation of cell proliferation. We discovered that SCIN knockdown significantly downregulated epidermal growth factor receptor (EGFR) protein expression, and inhibited epidermal growth factor (EGF)‐mediated cell proliferation and activation of the downstream mitogen‐activated protein kinase kinase (MEK)/extracellular signal‐regulated kinase (ERK) signalling pathway. Silencing of SCIN promoted apoptosis in two cell lines (PC‐3 and DU145), inhibited B‐cell lymphoma‐extra‐large (Bcl‐xl) expression and activated caspase signalling. Furthermore, *in vivo* studies showed that SCIN deletion slowed tumour growth and decreased EGFR expression. Thus, we conclude that SCIN promotes prostate cancer cell survival by stabilising EGFR and MEK/ERK signalling.

**Abbreviations**
7‐AAD: 7‐aminoactinomycin D
Bcl‐xl: B‐cell lymphoma‐extra‐large
CRPC: castration‐resistant prostate cancer
EGF: epidermal growth factor
EGFR: epidermal growth factor receptor
ERK: extracellular signal‐regulated kinase
FasLG: Fas ligand
GAPDH: glyceraldehyde 3‐phosphate dehydrogenase
GEO: gene expression omnibus
MEK: mitogen‐activated protein kinase kinase
MTT: 3‐(4,5‐dimethylthiazol‐2‐yl)‐2,5‐diphenyltetrazolium bromide
NEAA: non‐essential amino acid
PARP: poly‐ADP ribose polymerase
SCIN: scinderin
shCon: control shRNA
shRNA: short hairpin RNA
shSCIN: SCIN shRNA

Androgen deprivation therapy is the first choice for the treatment of metastatic prostate cancer 1. However, despite initial responses, the majority of patients eventually progress to castration‐resistant prostate cancer (CRPC) 2. The death of patients occurs because of bone, retroperitoneal lymph node and pelvic metastasis 3. Metastasis affects the integrity of epithelial cells, and tight junctions and adherent junctions are key regulators. The structure and organisation of these junctions depend on their association with the underlying cytoskeleton, which is the subject of complex regulation involving numerous structural, scaffolding and signalling molecules 4, 5. However, studies of cytoskeletal proteins in prostate cancer have not fully been reported.

Actin polymerisation was reported to partly mediate disodium pentaborate decahydrate‐induced cytotoxicity 6. Moreover, microtubule‐targeting chemotherapeutic agents are used in combination with anti‐androgen strategies to increase the survival rate of patients with advanced CRPC 7. These research studies suggest that the cytoskeleton could serve as a therapeutic target in advanced prostate cancer.

Previously, we reported a positive association in prostate cancer between poor differentiation status and scinderin (SCIN) 8, which is drastically upregulated in this condition. SCIN is a member of the calcium‐dependent gelsolin superfamily of actin severing and capping proteins that control actin organisation. SCIN protein comprises six homologous domains (namely A1–A6), and X‐ray crystal structure analysis has shown that calcium binding to the N terminus of SCIN dominates the activation process, which exposes the F‐actin binding site on A2 9. In agreement with our report, SCIN expression levels were shown to correlate with poor overall survival in patients with gastric cancers, and silencing of SCIN effectively suppressed the migratory and invasive capabilities and tumourigenicity 10. SCIN knockdown upregulated the expression of E‐cadherin and decreased that of N‐cadherin and β‐catenin in the SGC7901 gastric cell line 11. Silencing of SCIN also inhibited the proliferation of human lung carcinoma cells 12. However, the anti‐prostate cancer mechanisms of SCIN are still not fully understood.

In the present study, we investigated the role of SCIN in the regulation of cancer cell proliferation and its underlying mechanism by analysing related signalling pathway molecules.

## Material and methods

### ONCOMINE database analysis

The mRNA level of SCIN in prostate cancer was determined by analysing data in the ONCOMINE database (http://www.oncomine.org), which is a publicly available online human cancer microarray database. In this study, the *t* statistic was used to compare the cancer and normal control specimens using oncomine to generate a *P* value. The fold change and *P* value were set at 2 and 0.05, respectively.

### Cell lines and culture conditions

Two CRPC cell lines, PC‐3 and DU145, were purchased from Cell Bank of Chinese Academy of Science (Shanghai, China) and characterised as androgen‐independent and castrate‐resistant prostate cancer cell lines. DU145 cells have been reported to lack androgen receptor expression. Therefore, the two cell lines were selected for further studies. They were cultured in Ham's F‐12 medium (Thermo Fisher Scientific, Waltham, MA, USA) supplemented with 10% FBS (Biowest, Nuaillé, France) and 1% non‐essential amino acids (NEAA, HyClone, South Logan, UT, USA). All cell lines were maintained at 37 °C under a humidified atmosphere of 5% CO_2_.

### Plasmid DNA construction

The construction steps of the plasmid used in this study were described in our previous report 8.

### Cell transduction, RNA isolation, and quantitative reverse transcription polymerase chain reaction

PC‐3 and DU145 cells were both seeded at a density of 3000 cells per well in six‐well plates and allowed to adhere overnight. After transfection for 96 h, the cells were washed with PBS, and total RNA was isolated using a standard Trizol‐based technique. The specific primers for human SCIN were 5′‐TGCTGCCATCTTCACTGTTC‐3′ (sense) and 5′‐TGTAGGAGCCTCTTGGCTGT‐3′ (antisense), and those for β‐actin primers were 5ʹ‐GTGGACATCCGCAAAGAC‐3ʹ (sense) and 5ʹ‐ AAAGGGTGTAACGCAACTA‐3ʹ (antisense). SCIN expression level was normalised to that of β‐actin and then compared to the controls using the

$$2{-}^{\Delta \Delta C_{T}}$$

formula.

### Western blotting

Cells were washed, and the total protein was isolated using 100 μL radioimmunoprecipitation assay (RIPA) buffer (Beyotime, Shanghai, China). Then, each sample was loaded and separated using 10% SDS/PAGE and transferred to a polyvinylidene fluoride membrane (Millipore, Billerica, MA, USA). After blocking the membrane with 5% non‐fat milk, target proteins were detected using the following antibodies: anti‐SCIN, anti‐epidermal growth factor receptor (EGFR) anti‐B‐cell lymphoma‐extra‐large (Bcl‐xl), anti‐caspase 9, anti‐cleaved caspase 3, anti‐cytochrome *c* (all 1 : 1000; Proteintech, Rosemont, IL, USA), anti‐ glyceraldehyde 3‐phosphate dehydrogenase (GAPDH, 1 : 500 000; all Proteintech), anti‐mitogen‐activated protein kinase kinase (MEK), anti‐phosphorylated (p)‐MEK (both 1 : 1000; SAB, College Park, MD, USA), anti‐extracellular signal‐regulated kinase (ERK), anti‐p‐ERK (both 1 : 3000; Santa Cruz Biotechnology, Dallas, TX, USA), and anti‐Akt (1 : 1000; Cell Signaling Technology, Danvers, MA, USA), anti‐p‐AKT, anti‐poly‐ADP ribose polymerase (PARP; both 1 : 1000; Cell Signaling Technology). The appropriate horseradish peroxidase‐conjugated secondary antibody (Santa Cruz Biotechnology) was used, and the positive bands were detected using an ECL Plus kit (GE Healthcare, Little Chalfont, UK) and exposed to autoradiographic film (Kodak, Tokyo, Japan).

### MTT assay

To assess the effects of SCIN on epidermal growth factor (EGF)‐stimulated cell survival, all the cells including the short hairpin control (shCon) and short hairpin SCIN (shSCIN) cells were resuspended and seeded at 3000 cells per well in 96‐well plates with five replicates and treated with the indicated concentrations. The cells were stimulated with 50 ng·mL^−1^ EGF and then 3‐(4,5‐dimethylthiazol‐2‐yl)‐2,5‐diphenyltetrazolium bromide (MTT) was added at a concentration of 5 mg·mL^−1^ in 20 μL (M2128; Sigma‐Aldrich, St Louis, MO, USA) for the indicated time. The absorbance at 595 nm was measured using a spectrophotometer (BioTek Epoch, Winooski, VT, USA), and proliferation curves were constructed.

### Annexin V apoptosis assay

The annexin V/7‐aminoactinomycin D (7‐AAD) double staining kit (Keygen, Nanjing, Jiangsu, China) was used to detect and quantify apoptotic cells according to the manufacturer's instructions using flow cytometry. The apoptotic cells were analysed using a Gallios flow cytometer (Beckman Coulter, Indianapolis, IN, USA) and the data were analysed using the flowjo software (FlowJo, LLC, Ashland, OR, USA).

### *In vivo* tumourigenicity assays

Four‐week‐old male BALB/c (nu/nu) mice were purchased from SLRC Laboratory Animal Co., Ltd, (Shanghai, China) and divided into two groups of five mice each. The animal experiments were conducted following protocols approved by the Ethics Committee of Fuzhou General Hospital.

PC‐3 cells were suspended in PBS and subcutaneously injected into the axilla of each mouse at 5 × 10^6^ cells in a volume of 100 μL. The tumour volumes were calculated using the following formula: 0.4 × *L* × 2*W*, where *L* and *W* are the length and width of the tumours, which were measured every 3 days using callipers. At the end of the study, the tumours were excised and weighed.

### Statistical analysis

The data were analysed using prism 5.0 software (GraphPad Software, La Jolla, CA, USA). The data are presented as means ± standard deviation (SD). The differences between two normally distributed groups were estimated using Student's *t* test. *P* < 0.05 was considered statistically significant.

## Results

### Clinical significance of SCIN overexpression

**Figure 1:**
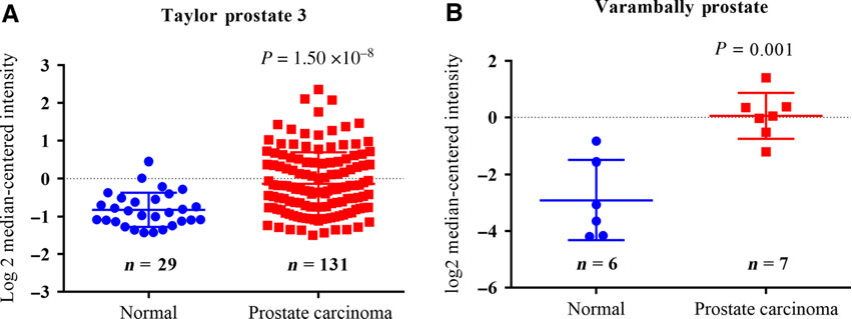
SCIN is elevated in prostate cancer samples compared to that in normal prostate tissue in (A) http://www.ncbi.nlm.nih.gov/geo/query/acc.cgi?acc=GSE21034 (Taylor Prostate 3 dataset) and (B) http://www.ncbi.nlm.nih.gov/geo/query/acc.cgi?acc=GSE3325 (Varambally Prostate dataset).

Our previous study showed that the SCIN protein level was negatively correlated with the differentiation status in prostate cancer 8. Poorly differentiated prostate cancer tissues show a high proportion of SCIN‐positive cells. In this study, our analysis of the gene expression omnibus (GEO) public database including http://www.ncbi.nlm.nih.gov/geo/query/acc.cgi?acc=GSE21034 (Taylor Prostate 3 dataset) 13 and http://www.ncbi.nlm.nih.gov/geo/query/acc.cgi?acc=GSE3325 (Varambally Prostate dataset) 14 further indicated that SCIN is generally elevated in prostate cancer samples compared to that in normal prostate tissue (Fig. 1).

### Depletion of SCIN decreases EGFR protein level and MEK/ERK signalling *in vitro*

**Figure 2:**
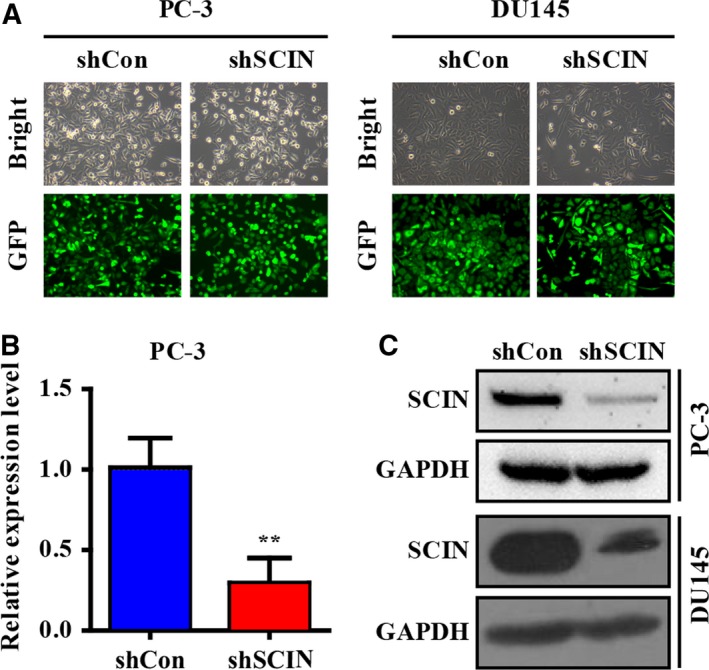
Efficiency of SCIN knockdown. (A) Representative graphs of bright and GFP fluorescence fields in shCon and shSCIN groups. (B,C) mRNA and protein levels of SCIN in shCon and shSCIN groups.

Our previous result showed that depletion of SCIN leads to PC‐3 cell arrest at the G1 phase and inhibition of cell proliferation, and the present study showed that SCIN knockdown reduced EGFR protein expression and altered the downstream MEK/ERK signalling pathway. First, SCIN was knocked down using a lentiviral‐mediated short hairpin RNA (shRNA) technology. Figure 2A shows representative graphs of the bright and GFP fluorescence fields in the control shRNA (shCon) and shSCIN groups of both PC‐3 and DU145 cell lines and indicates that the transfection efficiency in the two groups was > 90%. Then, the SCIN mRNA and protein levels were examined. As expected, the mRNA and protein levels significantly decreased in the shSCIN group compared with those in the shCon group in the two cancer cell lines (Fig. 2B,C).

**Figure 3:**
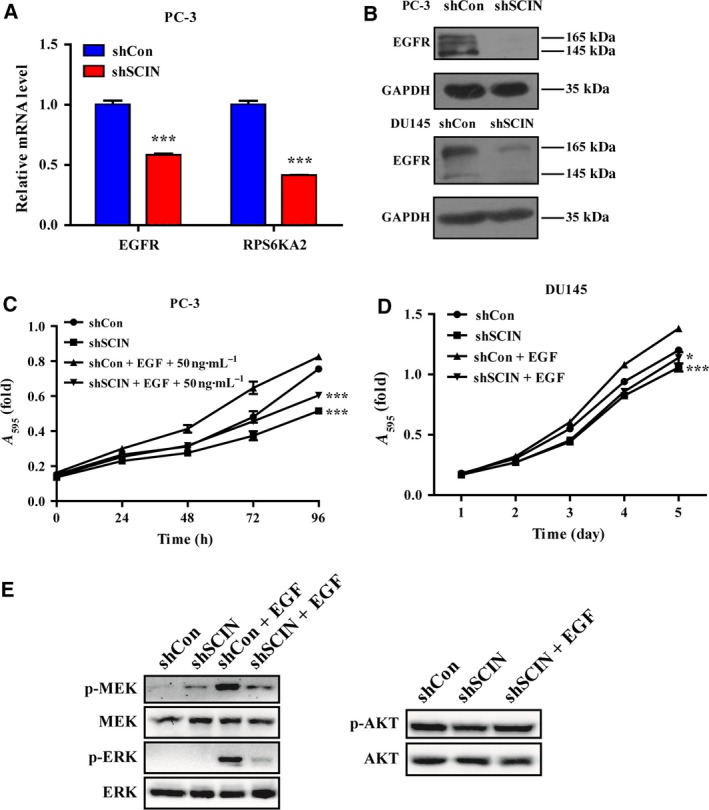
SCIN knockdown decreased EGFR, and phosphorylated ERK hampered EGF‐induced cell growth. (A) Relative expression of EGFR and RPS6KA2. (B) EGFR protein level in the two groups. (C,D) Cell growth curve detected using MTT assay. (E) MEK/ERK and Akt protein were detected using western blotting.

To assess whether other molecules in the prostate cancer cell were affected by the expression of SCIN, we preliminarily used a gene expression chip to screen PC‐3 cells (unpublished data) and found that the EGFR and RPS6KA2 expression was regulated by SCIN. A quantitative reverse transcription polymerase chain reaction analysis showed that the expression levels of EGFR and RPS6KA2 in the shSCIN group decreased (Fig. 3A), indicating that EGFR and its downstream MEK/ERK signalling may be influenced by SCIN. Therefore, we examined the expression level of EGFR protein in PC‐3 and DU145 cells and found it was significantly lower in the shSCIN group than it was in the control group (Fig. 3B). EGFR activation by EGF promotes cell proliferation and survival. The MTT results showed that 50 ng·mL^−1^ EGF significantly promoted the proliferation of shCon cells but not shSCIN cells (Fig. 3C,D). Furthermore, EGF upregulated the phosphorylation of MEK (p‐MEK) and ERK (p‐ERK) in shCon cells, whereas SCIN knockdown diminished the stimulatory effect of EGF on the MEK/ERK pathway (Fig. 3E). However, SCIN knockdown does not affect p‐Akt. These results indicate that SCIN deletion decreased the EGFR protein expression and inhibited the MEK/ERK signalling pathway activation.

### Depletion of SCIN induces cell apoptosis

**Figure 4:**
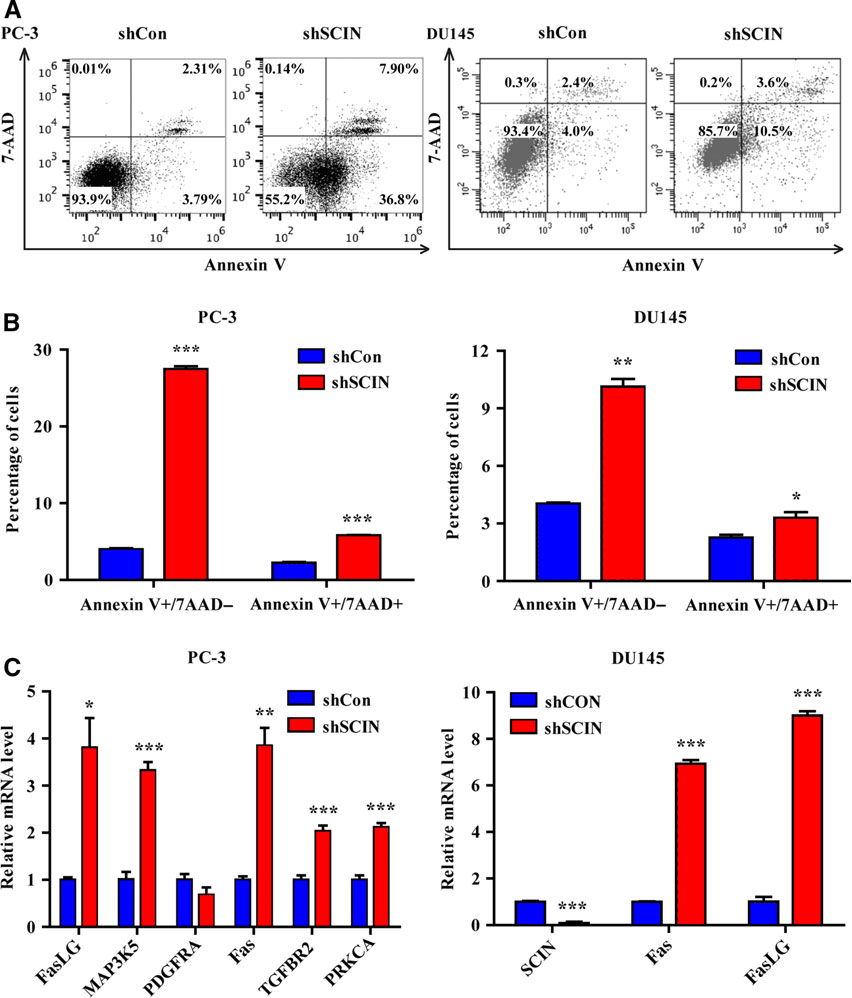
SCIN knockdown induced cell apoptosis. (A) Apoptosis of PC‐3 and DU145 cells were determined by Annexin V‐FITC and 7‐AAD staining. (B) Percentage of early and late apoptosis cells. (C) Fas and FasLG were increased in shSCIN group.

We analysed the ratio of apoptotic PC‐3 and DU145 cells (Fig. 4A). Expectedly, the early and late apoptosis ratios (annexin V^+^/7‐AAD^−^ and annexin V^+^/7‐AAD^+^, respectively) in the shSCIN groups significantly increased compared with that in the shCon groups (Fig. 4B).

Additionally, analysis of the gene chip data revealed that several apoptotic pathway molecules were upregulated in the shSCIN group. The independent cell experiments showed that the expression levels of Fas and Fas ligand (FasLG) mRNA significantly increased in SCIN‐depleted PC‐3 and DU145 cells (Fig. 4C).

**Figure 5:**
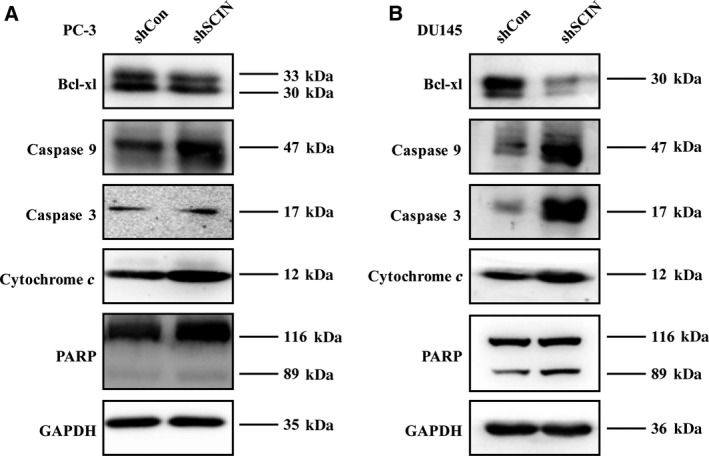
SCIN knockdown activated caspase 3 signalling pathway and induced cytochrome *c*.

We then analysed the expression of apoptosis‐related proteins and observed that cleaved cytochrome *c*, caspase 9, cleaved caspase 3 and cleaved PARP levels were elevated in the shSCIN groups compared with those in the shCon groups of both PC‐3 and DU145 cells (Fig. 5). Moreover, the protein level of Bcl‐xl decreased in the shSCIN groups of both cell lines. All the outcomes implied that the downregulation of SCIN promoted the apoptosis of prostate cells by regulating the expression of apoptosis‐related proteins.

### Loss of SCIN inhibits glioma growth *in vivo*

**Figure 6:**
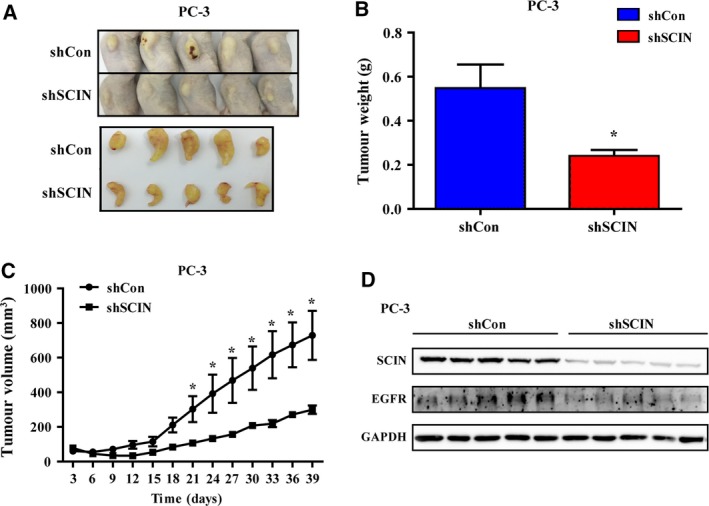
SCIN knockdown slowed tumour growth *in vivo*. (A) Image of mouse tumours. (B,C) Tumour weight and volume of the two groups. (D) EGFR and SCIN protein level of each tumour sample.

After subcutaneous implantation of PC‐3 cells into BALB/c (nu/nu) mice, we further evaluated the growth rates of prostate cells after knockdown of SCIN versus shCon. The results showed that the decreased expression of SCIN dramatically reduced the growth of tumours *in vivo* as indicated by the tumour weight (Fig. 6A,B) and volume (Fig. 6C). Moreover, the EGFR protein level was downregulated by silencing of SCIN in all tumour tissues (Fig. 6D). The results indicate that downregulation of SCIN inhibited tumour growth and the effect was mediated by EGFR *in vivo*.

## Discussion

In the present study, we demonstrated that SCIN, which is significantly overexpressed in prostate carcinoma, acted as a tumour growth promoter in prostate cancer. This phenomenon was evidenced by the SCIN depletion‐induced *in vitro* cell apoptosis and inhibition of *in vivo* tumour growth of CRPC cells. We also showed that loss of SCIN decreased the EGFR protein level and dampened the EGF‐stimulated MEK/ERK signalling pathway.

Scinderin is an important regulator of F‐actin organisation, and F‐actin is important in regulating the integrity of intercellular junctions in human epithelial cells, as reported by previous studies 4, 15. In our study, we demonstrated that loss of SCIN decreased the EGFR protein level significantly. Since the actin cytoskeleton limits intramembranous mobility and prevents its endocytosis from the plasma membrane 4, we hypothesised that the decrease in EGFR protein might be partially regulated by endocytosis. Moreover, the F‐actin‐binding protein Abp1 links the actin cytoskeleton to dynamin, a GTPase that supports endocytosis 16. In addition, the actin cytoskeleton is important for endocytosis 17. Similarly, F‐actin polymerisation stabilises E‐cadherin at epithelial junctions 15, indicating that the decrease in EGFR expression may have been mediated by F‐actin depolymerisation and endocytosis.

The actin cytoskeleton also acts as a scaffold for the organisation of the translation machinery components, and its perturbation dramatically reduces global protein synthesis in mammalian cells 18. Our results showed that loss of SCIN decreased the EGFR protein level but not that of total MEK or total ERK proteins, indicating that SCIN deletion did not perturb the global protein synthesis via the actin cytoskeleton. Furthermore, these findings may further support the hypothesis that endocytosis mediates EGFR‐selective degradation, and further studies of this phenomenon are warranted.

In several carcinomas, a high level of EGFR is associated with resistance to chemotherapy and has been linked to poor prognosis 19, 20. Activation of EGFR signalling is responsible for the bone metastasis and resistance to anti‐androgen therapy in prostate cancer 21, 22. Furthermore, EGFR inhibitors can induce apoptosis by altering the expression of specific Bcl‐2 family proteins 23. In the present study, downregulation of EGFR by shSCIN promoted cell apoptosis, which was evidenced by the decreased expression of the anti‐apoptotic protein Bcl‐xl and increased caspase 9 pathway signalling. Furthermore, EGFR downstream MEK/ERK signalling has been reported to upregulate the expression of the anti‐apoptotic protein BCL‐2 and inhibit the caspase cascade, leading to a decrease in cleaved caspase 3 24. Another research study reported that knockout of reticulocalbin‐2, a protein that interacts with EGFR, in hepatocellular carcinoma cells not only inhibited activation of the EGFR–ERK pathway but also suppressed cell proliferation under conditions of prolonged exposure to EGF 25. Collectively, EGFR proteins, whose levels are regulated by the actin‐regulating protein SCIN, are involved in cell apoptosis through the caspase 9 signalling pathway and MEK/ERK activity.

In conclusion, the evidence provided by this study supports that EGFR protein levels were selectively downregulated by loss of SCIN function. SCIN deletion diminished the EGF signal as evidenced by the phosphorylation of MEK and ERK and increased apoptosis of PC‐3 and DU145 cells. Pro‐apoptosis proteins were upregulated, including Fas, FasLG, cytochrome *c*, caspase 9, caspase 3 and PARP. Whether EGFR is degraded by endocytosis remains to be investigated. Nevertheless, our studies of SCIN and its functions may reveal novel strategies for prostate cancer management.

## Author contributions

DW and WW conceived and designed the project, WS, HZ and SY acquired the data, XL, HZ and TZ analysed and interpreted the data, DW and WW wrote the paper.
